# MicroRNA Profiling of CSF Reveals Potential Biomarkers to Detect Alzheimer`s Disease

**DOI:** 10.1371/journal.pone.0126423

**Published:** 2015-05-20

**Authors:** Johannes Denk, Kai Boelmans, Christine Siegismund, Dirk Lassner, Sönke Arlt, Holger Jahn

**Affiliations:** 1 Department of Psychiatry and Psychotherapy, University Hospital Hamburg-Eppendorf, Hamburg, Germany; 2 Institute for Cardiac Diagnostics and Therapy (IKDT), Berlin, Germany; Deutsches Krebsforschungszentrum, GERMANY

## Abstract

The miRBase-21 database currently lists 1881 microRNA (miRNA) precursors and 2585 unique mature human miRNAs. Since their discovery, miRNAs have proved to present a new level of epigenetic post-transcriptional control of protein synthesis. Initial results point to a possible involvement of miRNA in Alzheimer’s disease (AD). We applied OpenArray technology to profile the expression of 1178 unique miRNAs in cerebrospinal fluid (CSF) samples of AD patients (n = 22) and controls (n = 28). Using a Cq of 34 as cut-off, we identified positive signals for 441 miRNAs, while 729 miRNAs could not be detected, indicating that at least 37% of miRNAs are present in the brain. We found 74 miRNAs being down- and 74 miRNAs being up-regulated in AD using a 1.5 fold change threshold. By applying the new explorative “Measure of relevance” method, 6 *reliable* and 9 *informative* biomarkers were identified. Confirmatory MANCOVA revealed *reliable* miR-100, miR-146a and miR-1274a as differentially expressed in AD reaching Bonferroni corrected significance. MANCOVA also confirmed differential expression of *informative* miR-103, miR-375, miR-505#, miR-708, miR-4467, miR-219, miR-296, miR-766 and miR-3622b-3p. Discrimination analysis using a combination of miR-100, miR-103 and miR-375 was able to detect AD in CSF by positively classifying controls and AD cases with 96.4% and 95.5% accuracy, respectively. Referring to the Ingenuity database we could identify a set of AD associated genes that are targeted by these miRNAs. Highly predicted targets included genes involved in the regulation of tau and amyloid pathways in AD like MAPT, BACE1 and mTOR.

## Introduction

Alzheimer`s disease (AD) is a neurodegenerative, progressive disorder, which primarily affects people over the age of 65 [1]. Individuals suffer from memory deficiencies and other cognitive impairments as a result of synaptic dysfunction and neuronal decay. The development of preventive or curative therapeutic options as well as the establishment of favorable clinical biomarkers for (early) diagnosis and treatment efficacy is a permanent issue [2]. In spite of well-established diagnostic criteria such as traditional guidelines from the National Institute of Neurological Disorders and Stroke–Alzheimer’s Disease and Related Disorders Association (NINCDS-ADRDA), sensitivity and specificity of AD diagnosis is still lower than desirable. Amyloid-β (Aβ_1–42_), total-tau (tau) and phospho-tau (p-tau) are currently used as suitable cerebrospinal fluid (CSF) biomarkers to detect AD [3]. These proteins are components of the pathogenic hallmarks of AD, amyloid plaques and neurofibrillary tangles, and can be reliably measured in CSF if meticulous collection procedures are applied [4]. Another issue is the relatively less conclusive diagnosis of AD in contrast to related dementia pathologies such as vascular dementia, frontotemporal lobe dementia (FTLD), or Lewy body dementia.

It is commonly accepted that the late onset form of AD, which occurs after the age of 65, and accounts for about 90% of all AD cases, develops within complex interactions of multiple risk factors including genetic components, environmental influences and epigenetic mechanisms, making the identification of novel and informative biomarkers a challenging task [5, 6]. In the last decade, it has become increasingly clear that epigenetic mechanisms, such as DNA methylation, RNA editing or RNA interference considerably contribute to the development and course of AD pathophysiology [7]. RNA interference, especially, may offer potential for new diagnostic and therapeutic options for treatment of AD [8].

Central to this epigenetic process are miRNAs, a subclass of small noncoding RNAs, which are transcribed from either intra- or intergenic regions modulating gene expression post-transcriptionally by targeting mRNAs for cleavage or translational repression via base complementarity [9]. Their significant role in the proliferation, differentiation, function and maintenance of neuronal cells has already been demonstrated in several experimental systems [10]. Moreover, they are specifically expressed in neurons where they are suggested to function in synapse formation [11], synapse plasticity [12] and the differentiation of neurites [13]. The potential role that differentially expressed miRNAs may play in AD pathophysiology was first demonstrated by Lukiw (2007) [14] in hippocampal tissue and by Cogswell *et al*. (2008) [15], who studied miRNA expression changes in CSF and regions of the brain most affected by AD pathology. Furthermore, Hébert *et al*. (2008) showed that a loss of the miR-29a/b-1 cluster correlates with increased beta-secretase (BACE1) activity in Alzheimer’s disease pointing to a potential causative association [16]. Pathogenetically, it is suggested that elevated levels of BACE1 expression and activity might initiate or accelerate AD pathophysiology contributing to accumulated amyloid peptides [17]. In addition, Wang *et al*. (2008) reported that a change in neuronal miR-107 expression, which also targets BACE1, could contribute to the pathogenesis of AD [18]. Liu *et al*. (2012) provided strong evidence in AD SAMP8 (senescence-accelerated mouse prone 8) mice models, which have age-related learning and memory deficits, that miR-16 can regulate amyloid-precursor protein (APP) in vivo and that abnormally low expression of miR-16 levels potentially lead to APP accumulation [19].

Hence, miRNAs may provide valuable insight into the cellular mechanisms by which AD related genes are expressed or inhibited, thus improving the current understanding of cause or consequence of the disease progression at molecular level. They are considered as extremely stable [20, 21] and, owing to their function as regulators of gene expression, as well as their presence as circulating molecules in various body fluids [22], may arguably carry promise as biomarkers [23–25]. When measuring circulating miRNAs in neurodegenerative diseases such as AD, CSF is the best material, beside brain tissue, for pathological assessment and the identification of informative signals. Low RNA content in CSF, limited sample size and methodological problems accompanied by low detection limits have led to the production of conflicting results [26, 27]. Nevertheless, recent advances in technology and the development of guidelines may now facilitate research in this field. In our case, the entire qPCR protocol was performed on the basis of the MIQE guidelines (Minimum Information for Publication of Quantitative Real-Time PCR Experiments) [28] to reduce technical variability and to provide sufficient experimental detail to increase data transparency and validity. We profiled the expression of 1178 unique mature miRNAs (miRBase, version 14) in a patient cohort comprised of AD cases (n = 22) and a set of disease controls (n = 28) in human CSF drawn in a naturalistic approach from patients presenting to our memory clinic. Testing against disease controls instead against healthy probands is in our view a better way to differentiate towards AD specific changes in the miRNA signature.

## Materials and Methods

### Patient data and CSF

We measured the expression of miRNAs in CSF samples of a total of 50 probands by OpenArray RT-qPCR. The study cohort consisted of a naturalistic control group (n = 28) including cognitively healthy test subjects (n = 5), patients with normal pressure hydrocephalus (NPH) (n = 2), patients with FTLD (n = 9) and patients with cognitive impairment due to affective disorders or vascular disease (n = 12). This control group was compared to a group of AD cases (n = 22) composed of patients with probable AD (mild late onset AD) (n = 19) and mild cognitive impairment due to AD (n = 3). The groups were stratified for gender. Patients selected in this study referred to the memory clinic of the University Hospital Hamburg-Eppendorf. All patients underwent a diagnostic work-up and were diagnosed according to ICD-10 [29] and NINCDS-ADRDA criteria [30] to identify patients with AD involving new criteria and guidelines to diagnose AD supplanting the previous guidelines first published in 1984 [31–35]. Vascular dementia was diagnosed accordingly, FTLD (combining frontotemporal dementia and progressive non-fluent aphasia) according to the New Diagnostic Criteria for the Behavioural Variant of Frontotemporal Dementia [36, 37]. MCI diagnoses were made according to the criteria of Petersen [38]. Patients with mixed dementia etiologies were excluded. The present demographic data is summarized in Table 1.

**Table 1:** Summary of demographic data.

Variable	Control group	AD group	p
Number	28	22	
Gender (f/m)	14/14	13/9	ns
Age	61.0 ± 12.7	72.1 ± 8.5	0.0009
total tau [pg/ml]	308.9 ± 227.7	708.5 ± 282.9	< 0.0001
p-tau [pg/ml]	52.6 ± 28.5	92 ± 93.3	0.0003
Aβ1–42 [pg/ml]	719.9 ± 406.7	446.7 ± 164.1	0.0025
total RNA [ng/μl]	6.9 ± 3.4	6.7 ± 2.6	ns
purity [260/280 nm]	2.3 ± 0.7	2.3 ± 0.6	ns

Mean ± SD. Demographic data for the control- and AD group samples including cognitively healthy controls, NPH, FTLD as well as controls with cognitive impairment due to affective disorders or vascular disease and patients with probable AD and MCI due to AD. Given are numbers for each group and gender, the averages for age, total tau, p-tau, Aβ_1–42_, total RNA and RNA purity.

CSF was obtained by lumbar puncture in a sitting position according to standard procedures [39]. 4 ml CSF was collected into a polypropylene test tube for routine diagnosis as well as for further studies. CSF was free of blood contaminations and tested for hemoglobin. The sample was centrifuged (1600 g, 4°C, 15 min) and frozen within 30–40 min after the puncture and stored at -80°C until use. The CSF was at no time thawed/refrozen.

### Ethics Statement

Procedures were approved by the local ethics-committee of the Ärztekammer Hamburg. All patients and/or their relatives gave written informed consent. All clinical investigations have been conducted according to the principles expressed in the Declaration of Helsinki and have been carried out according to the international Good Laboratory Practice (GLP) and Good Clinical Practice (GCP) standards.

### Immunochemistry

The CSF levels of Aβ_1–42_, total tau, and phospho_181_-tau were measured using commercial ELISAs (Innogenetics, Ghent, Belgium) according to the manufacturer’s protocol. Cut-off values for AD suspicious biomarker concentrations were > 540 pg/ml for total tau, > 61 pg/ml for p-tau and < 240 + 1.186 x total tau pg/ml for Aβ_1–42_ values [3].

### RNA extraction, reverse transcription–qPCR and miRNA quantification

All qPCR experiments were designed and performed in compliance with the MIQE guidelines [28, 40]. We included a checklist to provide experimental detail related to each MIQE item (S1 Dataset).

Total RNA including small RNA was isolated using the mirVana PARIS Kit (Ambion, PN AM1556) following the manufacturer’s recommendations. In brief, the samples were homogenized in a denaturing lysis solution, spiked with kshv-miR-K12-1-5p (artificial miRNA) and subjected to an acid-phenol:chloroform extraction. After first separation of the two phases, an additional spiking with ath-miR159a cDNA was performed. Hereafter, the samples were purified on a glass-fiber filter and quantified using a Bioanalyzer 2100 (Agilent Technologies). Concentration and purity were measured using the Nanodrop ND1000 (Peqlab).

Total RNA was converted to cDNA using Megaplex stem-loop RT primer (Life Technologies, PN 4444750) for Human Pool A and B and custom RT primer for Pool C and D in combination with the TaqMan MicroRNA Reverse Transcription Kit (Life Technologies, PN 4366596). This allowed simultaneous cDNA synthesis of 377 unique miRNAs for each Pool A and B and 212 unique miRNAs for each Pool C and D. In brief, 3 μl of total RNA was supplemented with RT primer mix (10x), dNTPs with dTTP (100 mM), Multiscribe Reverse Transcriptase (50 U/μl), RT buffer (10x), MgCl_2_ (25 mM), and RNase inhibitor (20 U/μl) in a total reaction volume of 7.5 μl. Thermal-cycling conditions were as follows: 40 cycles at 16°C for 2 minutes, 42°C for 1 minute, and 50°C for 1 second, followed by reverse transcriptase inactivation at 85°C for 5 minutes.

The RT product (7.5 μl) was preamplified by using the TaqMan PreAmp Master Mix (Life Technologies, PN 4391128) and preamplification primers (Life Technologies, PN 4444750) in a 40 μl PCR reaction. For each pool of stem-looped RT primers in the cDNA reaction, a different pool of PreAmp Primers (Human Pool A and B resp. custom PreAmp primers Pool C and D) was used. Thermal cycling conditions were as follows: 95°C for 10 minutes, 55°C for 2 minutes, and 72°C for 2 minutes, followed by 16 cycles of 95°C for 15 seconds and 60°C for 4 minutes. 4 μl PreAmp product was diluted in 156 μl 0.1x TE-Buffer.

The performance of RNA extraction, RT-qPCR and preamplification was checked by running single quantitative PCRs including the assays for U6 snRNA, ath-miR-159a, and kshv-miR-K12-1-5p on a 7900HT Fast Real-Time PCR System (Life Technologies, Darmstadt, Germany). In brief, 1 μl of the diluted preamplified product was supplemented with 10 μl TaqMan Universal PCR Master Mix, No AmpErase UNG(2x), 1 μl individual TaqMan Assay (20x) and 8 μl aqua dest. Thermal cycling conditions were as follows: 95°C for 10 minutes, followed by 40 cycles at 95°C for 15 seconds and 60°C for 1 minute.

miRNA quantification was performed with the TaqMan OpenArray Human MicroRNA Panel according to the recommended protocol (TaqMan OpenArray MicroRNA Panels, PN 4461306) for the reactions A and B (Life Technologies, PN 4461104) on one array with in total 818 TaqMan assays and two custom OpenArray plates for reaction C and D (Life Technologies, PN 4461104) separately, each on an individual array with 212 miRNA assays. For each reaction A and B, 45 μl of PCR reaction mix containing 22.5 μl of TaqMan OpenArray Real-Time PCR Master Mix (Life Technologies, PN 4462159) and 22.5 μl 1:40 prediluted preamplified product were prepared. For each reaction C and D, 25 μl of PCR reaction mix containing 12.5 μl of TaqMan OpenArray Real-Time PCR Master Mix and 12.5 μl 1:40 prediluted preamplified product were prepared. 5 μl of each prepared master mix were loaded in one well of a 384-well plate several times to obtain a usable format for automatic pipetting. TaqMan OpenArray Human MicroRNA Panels and custom OpenArray plates were then automatically loaded using the AccuFill System (AccuFill System User Guide, PN 4456986). Up to 3 resp. 12 samples per OpenArray plate were cycled simultaneously on a Biotrove OpenArray NT Cycler (Life Technologies) using OpenArray Real-Time qPCR Analysis Software (v1.0.4) with a pre-assigned cycling program to calculate quantification cycle (Cq) defined as the number of cycles at which the fluorescence signal is significantly above the threshold.

The NormFinder algorithm was applied using GenEx software version 5.4.3 (MultiD) to identify reference genes. The arithmetic mean of their Cq values was calculated for normalization and was subtracted from all miRNAs of each pool to yield ΔCq values. Relative miRNA expression levels between test groups were calculated by using the 2^(-ΔΔCt)^ method [41]. Relative expression levels of individual miRNAs were presented as 2^(-ΔCt)^ in log2 scale.

### miRNA target predictions

MiRNA targets were predicted *in silico* by using the microRNA target filter tool implemented in Ingenuity Pathway Analysis (IPA) (Ingenuity Systems, www.ingenuity.com). Prediction confidence was set to experimentally observed and highly predicted targets. Disease filter was limited to “neurological” and “psychological”, i.e. psychiatric disorders. Species was set to human and only tissues and primary cell lines of the central nervous system and CNS cell lines were considered for filtering.

### Statistical analysis

We set Cq ≤ 34 as cut-off to define a miRNA as positive or as actively expressed. From all positive miRNAs in the sample population only those showing an occurrence frequency (FOC) of at least 3 in each of the considered two groups (n = 199 miRNAs and Tau, p-tau and Aβ_1–42_) were considered for statistical analysis. The subset was further divided into a set A of 59 **abundant** markers (FOC≥19 in the control- and ≥17 in the AD group) and a set B including 143 **less abundant** markers (FOC≤18 in the control- and ≤16 in the AD group). Finally, 202 potential markers were statistically evaluated:

We first applied the explorative ‘Measure of Relevance’ (MoR) method (Yassouridis *et al*., 2012) to the 202 potential markers in order to identify the most *informative* miRNAs (further called as “*informative* biomarkers”), i.e. miRNAs that can differentiate well between AD and control groups [42]. The explorative MoR method, which is based on a measure containing relevant information of the distribution form, location and dispersion parameters of the samples, enables reasonable reduction of data dimensionality without the need for test decisions, corrections of significance levels and other presumptions. Especially for our two samples that possess relative small sizes (28 and 22) compared to the large number (202) of potential markers, inferential statistics applied to all of them are not advisable, not only because of the commenced power weakness to detect significances after correction of the level of significance but also because the considered miRNAs (e.g. for miRNA families) will likely display dependencies to each other. Therefore, we decided to apply first the aforementioned explorative analysis for identifying the most *informative* miRNAs and thereafter to perform confirmatory statistical analysis only to them. This method works as follows:

In a two-sample problem each of the considered variables—irrespective of their abundance—is provided after suitable transformations and rank allocations with a positive number (measure of relevance) which is proportional to the capability degree of the variable to discriminate between the samples. The higher the discrimination capability of a variable, the more informative it is towards the two-sample problem. All attached MoR-values are then sorted in an ascending order (information chain) along which a critical value by means of a suitable algorithm (stop criterion) has to be determined. After the determination of the critical value all variables with corresponding MoR-values bigger than the critical value of the information chain are declared as *informative* variables. If the critical value is higher than all MoR-values, none of the variables is considered *informative*. We chose at least 0.57 as critical value as it corresponds to a medium to large effect size with effect sizes higher in set A compared to set B.

To further improve the results of the explorative analysis an additional reliability investigation by applying repeatedly the MoR method to randomly chosen smaller and different subgroups of the considered groups was performed. For receiving a sufficient number of such subgroups only those 59 potential markers with at least 17 positive signals in each group (set A) were considered for the reliability analysis. For the other 143 miRNAs (set B) the explorative analysis was restricted to a unique application of the MoR method. For the reliability analysis 800 different sub-samples were used with 15 probands randomly chosen from each group. Variables among the 800 repetitions proven to be *informative* with a relative frequency (RF) over 0.8 were also declared as *reliable* biomarker candidates (further called as “*reliable* biomarkers”) because they are able to distinguish very well between AD and control group at the exploratory level. The reliability investigation was performed twice: once without substitution and once by substitution of missing values with group mean. After identifying the *reliable* biomarker candidates of set A and the most *informative* variables of set B, inferential statistics followed by applying multivariate analyses of covariance (MANCOVA) with sex and age as covariates. Those miRNAs among the biomarker candidates, which revealed significant differences between the AD and control group after Bonferroni adjustments on the confirmatory level, were designated as significant biomarkers. To explore the discrimination power of the *informative* miRNAs from set A and set B some of their combinations were additionally subjected to a discriminant analysis. For testing associations between miRNA markers and predicted mRNA targets in amyloid and tau pathways according to IPA’s database, Pearson’s correlation coefficients were calculated and proved about significance. For testing significance in some demographic variables with metrical or non-metrical data structure, two-sided student t-tests and x²-test were applied, respectively. As nominal level of significance α = 0.05 was accepted and corrected according to the Bonferroni procedure, whenever post-hoc multiple tests have to be performed.

## Results

### CSF miRNA expression profile in the sample population

**Fig 1:**
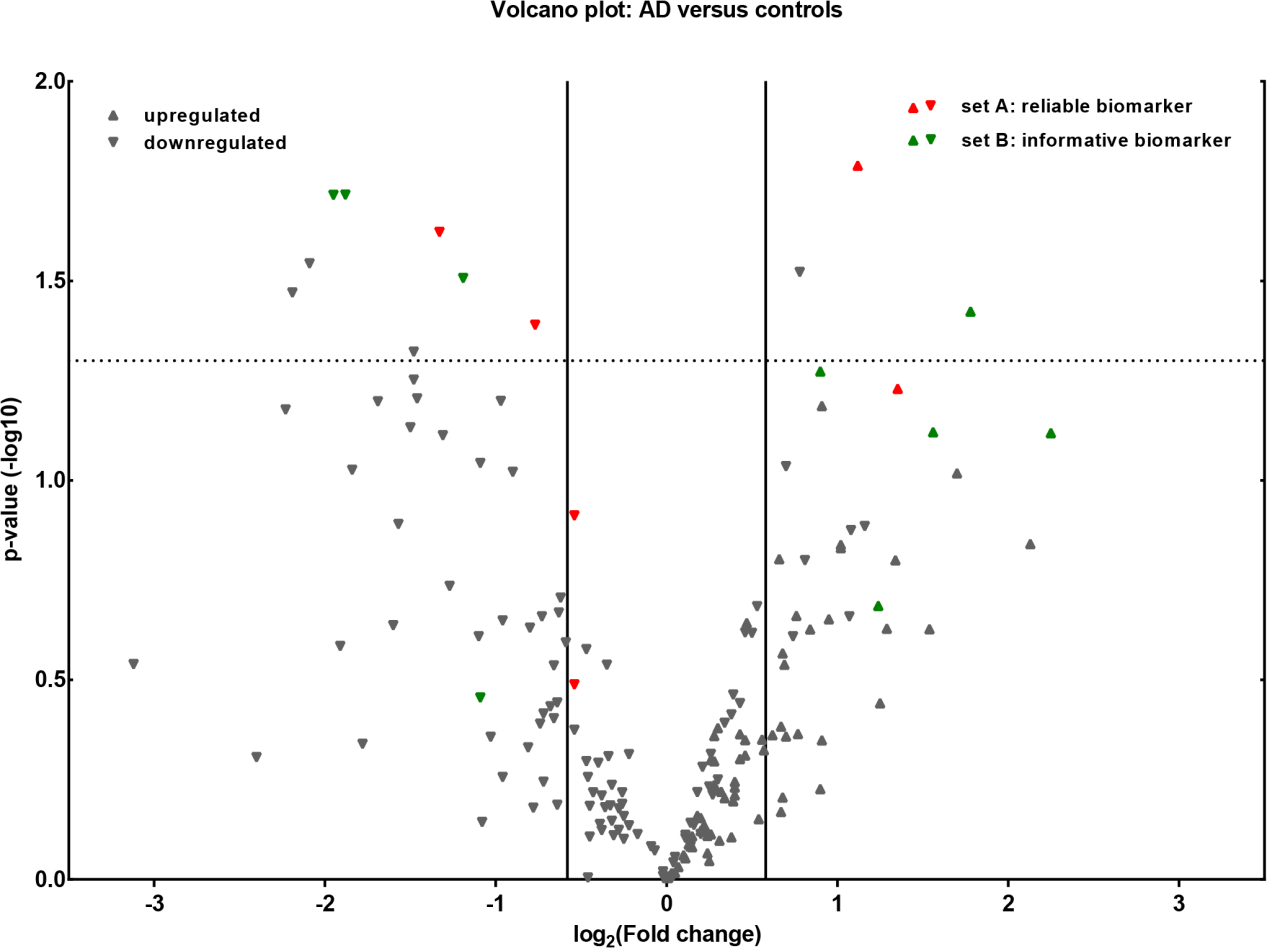
Volcano plot of group comparisons. Comparisons of 199 miRNAs assessed in OpenArray analysis of smallRNA isolated from CSF of patients with AD (n = 22) and controls (n = 28). The volcano plot displays the relationship between fold change and significance between the two groups, applying a student’s t-test. The y-axis depicts the negative log10 of p-values of the t-tests (the horizontal slider at 1.3 corresponds to a p-value of 0.05, a higher value indicates greater significance) and the x-axis is the difference in expression between the two experimental groups as log2 fold changes (vertical sliders indicate miRNAs as either up- or down regulated above a fold change of 1.5). Highlighted in green are the *most reliable* (6 abundant miRNAs from set A with RF≥0.8) and in red the *most informative* (9 less abundant miRNAs from set B above the critical MoR value d = 0.57) biomarkers according to the MoR-method (see Figs 2 and 3).

After profiling the expression of in total 1266 (1178 w/o controls) miRNAs (miRBase version 14) in CSF of 22 AD patients and 28 disease controls, 441 (380 in control- 359 in AD group) miRNAs were positively detected in our sample cohort (S2 Dataset). For 729 miRNAs we did not find detectable traces in CSF. This is an indication that at least 37% of the investigated miRNAs appear in CSF and are potentially active in the brain corresponding well with the fact, that about a third of the approximately 20.000 different genes that make up the human genome are active in adult brain [43]. Fig 1 displays only those miRNAs from set A (n = 56) and set B (n = 143) to illustrate only corresponding fold changes of potential markers that have been detected in both groups with an FOC of at least ≥ 3. All remaining miRNAs were not included in statistical analysis either due to low expression levels or low occurrence frequencies. When comparing AD cases with controls, 74 miRNAs were identified as down-regulated and 74 miRNAs up-regulated, using a fold change threshold ≥ 1.5. (Fig 1). Moreover, Fig 1 highlights 15 miRNAs that were identified as *reliable* or *informative* biomarkers by using the MoR-method.

A problem in miRNA studies is often the lack of suitable normalization procedures. For CSF no consensus exists. We identified mir-21, miR-24, miR-328, miR-99b, miR-let-7c and miR-1274B as not regulated between groups, and as potential reference genes for normalization of miRNA expression levels in CSF. The application of the explorative MoR method based on standardized differences of ranks works almost independent of data structures. However, normalization procedures are also necessary on the confirmatory level, i.e. in our case the subsequent MANCOVA analyses.

The difference in mean age between the two groups reached statistical significance (t-test, p<0.05) (Table 1), prompting us to define age as a covariate, although we did not find associations of miRNAs expression with age. Regarding sex, a possible second covariate, the two groups were stratified and therefore statistically significant differences between groups (x²-test, p = ns, Table 1) should not arise. However, similarly to some studies already reporting gender effects on some miRNAs for e.g. human brain tissue [44], we found gender specific differences for miR-106a, miR-17 and miR-320 in set A and miR-19a, miR-221, miR-532, miR-95 in set B. Therefore, we decided to control the results towards age and sex by considering these variables as covariates in the confirmatory MANCOVAs.

### Identification of potential biomarkers applying the “Measure of Relevance” method

#### Reliability analysis in set A

**Fig 2:**
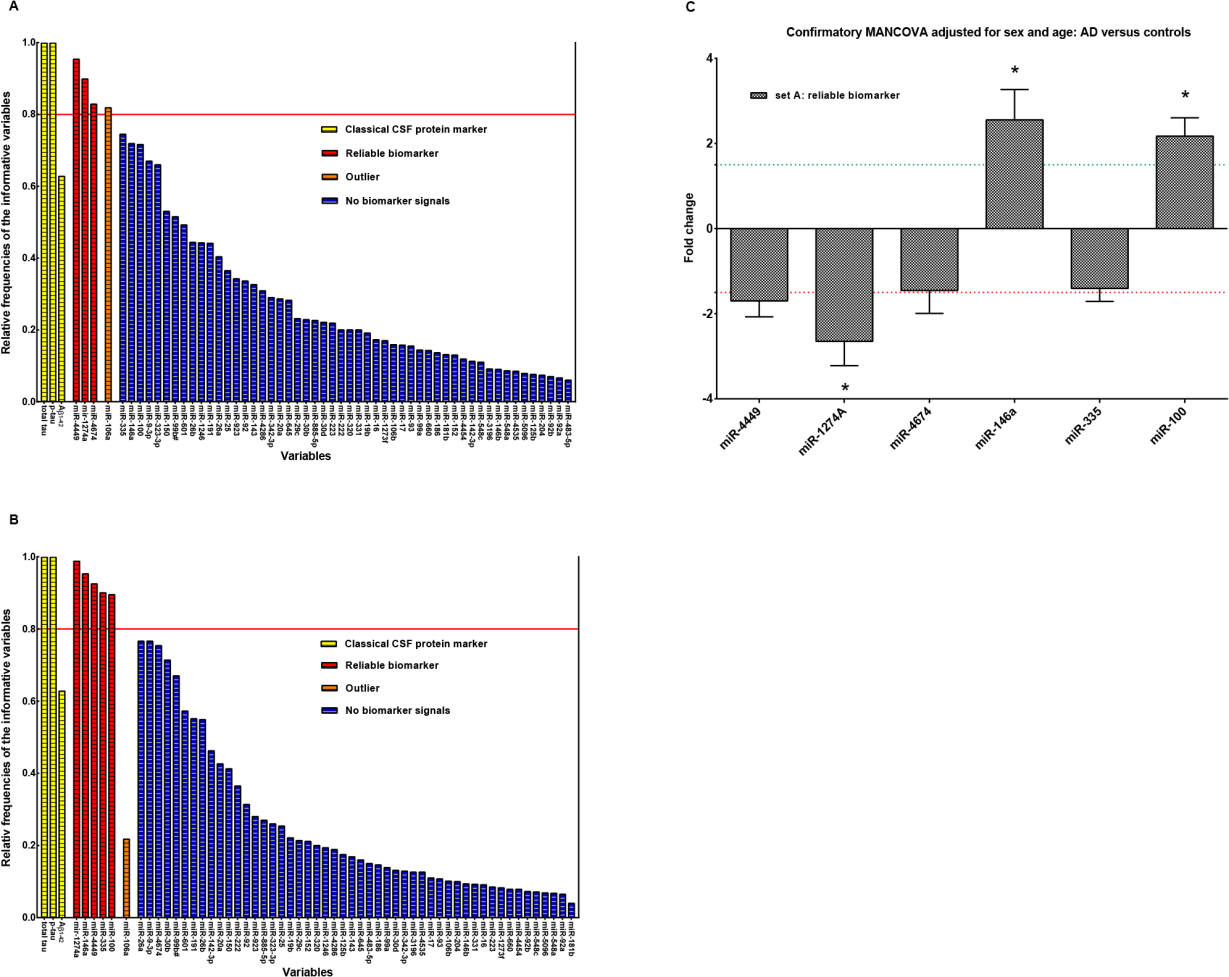
Reliability investigation. Plot of relative frequencies denoting for a miRNA how often among 800 MoR repeats with random subsamples of the original groups it has been crystallized as *informative* (original groups: controls n = 28, AD n = 22 subjects). (A) Relative frequencies of set A miRNAs with reliable biomarker candidates over the solid red line (RF = 0.8); (B) Relative frequencies of set A miRNAs with reliable biomarker candidates over the solid red line (RF = 0.8) after substitution by corresponding group means; (C) Bar diagram of the reliable biomarker signals of set A. Stars (*) over the bars point to significant p-values (MANCOVA, p < α*, where α* is Bonferroni corrected α = 0.05) and therewith to significant biomarkers.

As already reported for obtaining robust and better results in the explorative analysis a reliability investigation based on the MoR-method was performed on set A. This included 59 potential markers with n = 56 highly expressed (mean Cq 25.06) miRNA species with elevated FOC and CSF markers tau, p-tau and Aβ_1–42_. Without substitution of the missing values the reliability investigation identified among the 59 biomarker candidates in set A, miR-4449, miR-1274a, miR-4674 and miR-106a as *reliable* biomarker candidates with RF ≥ 0.8 threshold (Fig 2A). After substitution of missing values by group mean, miR-4449, miR-1274a, miR-146a, miR-335 and miR-100 were found as *reliable* candidates with RF ≥ 0.8 (Fig 2B). Interestingly, miR-106a that proved to be a *reliable* biomarker candidate without substitution after missing-values substitution lost this property. A possible explanation would be that after missing values substitution, which generally reduces the pooled variance between groups, the MoR-values of other miRNAs will be somewhat higher than of miR-106a and push the position of miR-106a below the critical MoR-value of the information chain. Classical CSF biomarkers total tau, p-tau and Aβ_1–42_ were also subjected to the reliability analysis as internal controls to validate the MoR algorithm of correctly identifying *reliable* biomarker candidates and to compare relative frequencies with miRNAs from set A. In this case, both, total tau as well as p-tau scored with RF = 1.0, confirming functionality of the MoR approach (Fig 2A and 2B). Interestingly, Aβ_1–42_ was not identified as a *reliable* biomarker (Fig 2A and 2B). This is probably due to the fact that Aβ_1–42_ protein levels vary widely across various dementia forms, again displaying that its degree of information as a single biomarker may not suffice in clinical routine diagnostics due to its low specificity [45]. The 6 *reliable* miRNA biomarker candidates from set A, tau and p-tau were subsequently subjected to MANCOVA after substitution of missing values by the corresponding group mean with age and sex as covariates in order to prove by inferential means the capability of these miRNAs to distinguish between the AD and control group. MANCOVA revealed a significant group effect [Wilks multivariate test of significance; F(8,39) = 8.79, sig of F < 0.00001]. Bonferroni correction pointed to a significant differential expression of miR-1274A, F(1, 46) = 16.58, p = 0.000, miR-100 [F(1, 46) = 7.85, p = 0.007], miR-146a [F(1, 46) = 4.78, p = 0.034] and naturally tau [F(1, 46) = 22.67, p = 0.000] and p-tau [F(1, 46) = 13.96, p = 0.001] between groups (Fig 2C). In this case, miR-1274A, miR-100 and miR-146a (Fig 2A and 2C and S3 Dataset) were confirmed as *reliable* and significant biomarkers. The covariates sex and age did not seem to exert significant effects on the considered miRNAs [Wilks multivariate test of significance; F(16,78) = 0.85, sig of F = 0.629]. IPA analyses predicted GRIN2A (miR-4449); IRAK3 (miR-4674); MAPT (miR-146a); ADAM19, BDNF (miR-335) and mTOR, TARDPB (miR-100) as targets of our deregulated miRNAs from set A (S4 Dataset).

#### *Informative* miRNAs in set B

**Fig 3:**
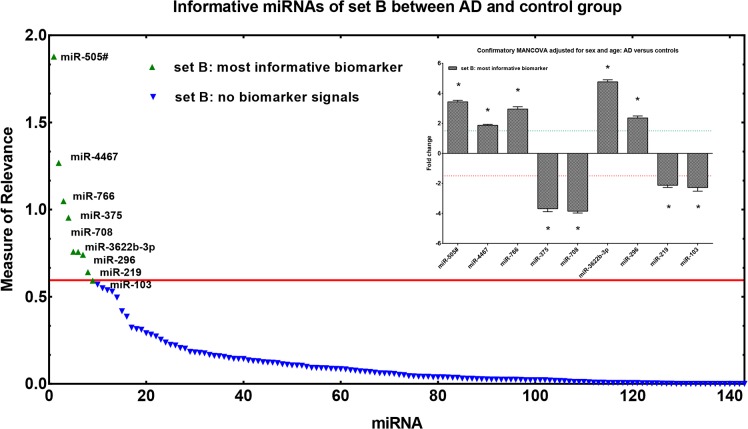
Measure of Relevance for miRNA expression data in CSF. Application of the MoR approach to miRNAs of set B for identifying relevant expression differences between controls and AD cases. In the control group n = 28 and in the AD group n = 22 subjects were examined. MoR-values over the red line are to be declared as *informative* (critical MoR-value for the *informative* designation d = 0.57). Bar diagram of the most *informative* biomarker signals of set B. Stars (*) over the bars point to significant p-values in MANCOVA (MANCOVA, p < α*, where α* is Bonferroni corrected α = 0.05).

Set B covered all moderately expressed (mean Cq 28.2) miRNAs (n = 143) with lower FOC and was exclusively subjected to a unique MoR analysis. Applying the MoR approach 9 out of the 143 potential miRNA biomarker candidates were identified as *informative* (Fig 3). The MoR plot illustrates the 9 *most informative* miRNAs, hsa-miR-505-5p, hsa-miR-4467, hsa-miR-766, hsa-miR-375, hsa-miR-708, hsa-miR-3622b-3p, hsa-miR-296, hsa-miR-219 and hsa-miR-103, each reaching a MoR value ≥ 0.57 (critical MoR value on the information chain). The 9 *informative* miRNAs were subsequently subjected to MANCOVA again with sex and age as covariates. After substitution of missing values by the corresponding group mean, MANCOVA revealed a significant group effect [Wilks multivariate test of significance; F(9,38) = 90.79, sig of F < 0.00001] for all *informative* miRNAs identified in set B. This effect was further shown to be highly significant for each individual marker by reaching Bonferroni corrected significance (Fig 3; univariate F-tests, p < 0.000). Furthermore, the covariates sex and age did not show a significant association with the miRNAs. Most relevant gene targets identified by IPA were BACE1, REST for miR-103, MAPT for miR-219 and CDK5R1 for miR-375 (S4 Dataset).

### miR-146a expression levels implicated in tau pathomechanism

**Fig 4:**
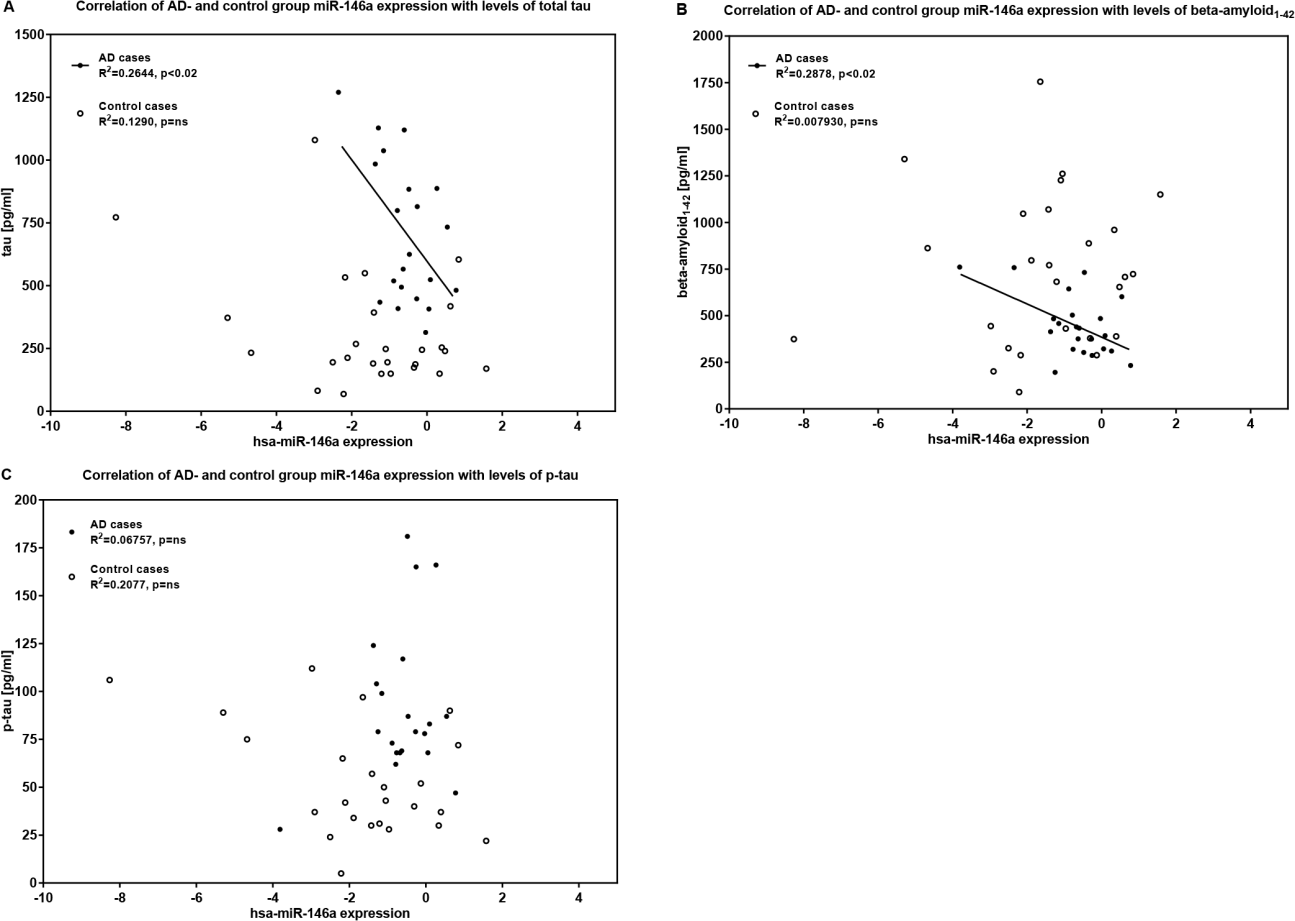
Scatter plot: Correlation of CSF miR-146a expression and levels of total tau, p-tau and Aβ_1–42_. Correlation significances were proven by Pearson correlation coefficients. Data were analysed applying Cq 32 as cut-off for miR-146a expression levels. Expression levels of miR-146a (2^-dCt log2) are inversely correlated with concentrations of total tau and Aβ_**1–42**_ in the AD group: (A) miR-146a expression levels vs. tau, AD cases: r = -0.5142, 98% CI -0.8065 to -0.01993, p = 0.0171; (B) miR-146a expression levels vs. Aβ_**1–42**_, AD cases: r = -0.5364, 98% CI -0.8121 to -0.06519, p = 0.0101. (C) No significant correlations were observed for concentrations of p-tau and miR-146a expression levels in the AD- and control group. Aβ_**1–42**_ = b-amyloid 42; CI = confidence interval; p-tau = phosphorylated tau.

Increased levels of tau protein and its phosphorylated derivate as well as decreased levels of extracellular Aβ_1–42_ peptides have been proven as markers to detect AD in CSF [1]. It remains still unclear to what extent these proteins contribute to AD pathogenesis and whether the expression of one protein explains the toxic effect of the other. We investigated correlations of our significant miRNA signals with these classical biomarkers. According to Ingenuity’s database, miR-146a is highly predicted to target the MAPT gene. MiR-146a expression levels were significantly upregulated in CSF of AD patients (Fig 2C) and showed a significantly inverse correlation with tau and Aβ_1–42_. Lower miR-146a expression levels were accompanied by higher levels of tau (AD cases: r = -0.5142, p = 0.0171) and Aβ_1–42_ (AD cases: r = -0.5364, p = 0.01), and vice versa in our AD group (Fig 4A and 4B). No significant correlation with concentrations of p-tau was observed in the AD group (Fig 4C). In the control group no significant correlations with miR-146a emerged. We also found significant correlations between miR-103 targeting BACE1 and both tau for the whole study sample (r = -0.4223, p = 0.045) and Aβ_1–42_ (r = 0.5980, p = 0.024) for the control group. MiR-375, which is thought to downregulate CDK5R1, was downregulated in our AD cases and correlated significantly with Aβ_1–42_ (r = 0.7481, p = 0.002).

### Classification

The combination of miR-146a and p-tau as biomarkers already allowed correct classification in 86.4% of all cases performing discriminant analysis. Using ROC curve analysis the combination showed an AUC of 0.64 for miR-146a and an AUC of 0.79 for p-tau (S5 Dataset). Another discriminant analysis performed on the most *reliable* biomarker miR-100 from set A (Fig 2B and 2C and S3 Dataset) and the most abundant miR-103 and miR-375 from set B (S2 Dataset and S3 Dataset) revealed for the two test groups a total correct classification rate of 96% after substitution of missing values, positively classifying controls and AD cases with 96.4% and 95.5% accuracy, respectively. ROC curve analysis showed an AUC of 0.72 (miR-100), an AUC of 0.87 (miR-103) and an AUC of 0.99 (miR-375) for this combination (S5 Dataset).

## Discussion

Due to its direct and intimate relationship with brain tissue we consider CSF a more suitable and informative material for the potential monitoring of neurophysiological changes in AD. To the best of our knowledge, we have profiled the largest number of unique miRNAs (n = 1178) in CSF from the largest AD/control (n = 22/28) sample cohort. We reported here that at least 37% of miRNAs are expressed in the human brain, i.e. 441 out of 1178 investigated miRNAs showed detectable traces pointing to transcriptional activity in CSF. This observation corresponds well with the fact that the highest expression of tissue specific miRNAs is also found in the brain [46]. Consistent with results of the Cogswell study [15], which looked at a smaller set of miRNAs, we also observed an even distribution of under- and overexpressed microRNAs in CSF of patients with AD compared to a control group. The number of detected miRNAs in our study was considerably higher than those reported in a recent miRNA profiling study by Frigerio *et al*. [47]. This is probably due to the different platform used and the inclusion of a preamplification step in our protocol. We applied OpenArray technology including a preamplification step on a larger set of patients (n = 50) to overcome some obstacles that may have caused contradicting results [48]. The preamplification step improves sensitivity and increases the number of detectable miRNAs without introducing a systemic bias in the estimation of miRNA expression [49]. However, we observed a great overlap of detected miRNAs in our dataset with those reported by Frigerio *et al*. [47].

For the first time, we applied a sophisticated and exploratory statistical approach (Measure of Relevance) to analyse miRNA expression data in order to identify potential biomarkers. The Measure of Relevance algorithm detected 15 *informative* miRNA markers in our CSF samples. Of those 15 candidates, 3 of 6 miRNAs from set A, on which a reliability analysis could be performed, were also inferentially confirmed at Bonferroni corrected significance by MANCOVA. Thus, miR-100, miR-1274a and miR-146a might be strong candidates for new AD biomarkers.

Beside its biomarker potential miR-100 could also be an interesting target for therapeutic interventions. Due to a high seed pairing stability and its CG dinucleotide rich seed site, miR-100 is supposed to have only few mRNA targets, among them mTOR (mammalian target of rapamycin) and TARDPB [50]. Recently, it was shown that reducing mTOR signalling increases lifespan. There is an association between mTOR and tau, which is linked to GSK3β and autophagy function. A reduction of mTOR signalling might alleviate pathologically increased tau phosphorylation [51]. While Caccamo *et al*. provided preclinical data indicating that reducing mTOR signalling may be a valid therapeutic approach for tauopathies, our results suggest that this salvage pathway may already be active in AD patients by up-regulation of miR-100 (fold change 2.17). Interestingly, we found on a trend-level a negative correlation of CSF miR-100 concentrations with CSF p-tau in our controls (r = -0.42, p = 0.065) but not in our AD samples (r = -0.0188, p = 0.941). This might point to a ceiling effect. Furthermore, miR-100 is up-regulated in the medial frontal gyrus of AD patients but not in hippocampus in analogy to the expected tau progression in AD, which could explain elevated CSF concentrations of miR-100 due to the release during atrophic processes [15].

An unexpected result was the identification of miR-1274a, which resembles a t-RNA and probably not a real miRNA, as significantly deregulated in AD, whereas miR-1274b was identified as a reference gene at the same time, demonstrating stable expression levels across our study population. According to annotated miRbase.org, the mature sequences of miR-1274 are considered as fragments of a Lys tRNA and are proposed to be endogenous retroviral elements [52]. It is reported that genes from human endogenous retroviruses have been detected as transcripts and proteins in the central nervous system, frequently in the context of neuro-inflammation. These elements have also been implicated in multiple sclerosis and other neurological diseases and should, according to our findings, be subject of further investigation [53].

MiR-146a is a brain-specific miRNA that is also associated with neuro-inflammation [54]. It is suggested that pro-inflammatory and innate immune system-associated factors play a role in pathways that drive the pathological AD process [55]. In line with results of Alexandrov (2012) we also observed significant increases of miR-146a expression in AD and proved miR-146a to be abundant in CSF [56]. Another study reported that miR-146a expression is induced by NF-kB and considered to downregulate complement factor h, an important repressor of the inflammatory immune response of the brain, which could explain differential expression in AD brain and relate neuro-inflammation to AD pathogenesis [57]. Analysing correlations of miRNA expression levels with clinical biomarkers (tau, p-tau and Aβ_1–42_) yielded a complex correlation pattern for miR-146a, which is also predicted to target MAPT in-silico (S4 Dataset). We found high miR-146a expression in our AD patients (fold change 1.81) and significant negative correlations of miR-146a with tau and Aβ_1–42_ levels, pointing to a possible inhibitory mechanism of miR-146a on tau production. Changes of miR-146a concentrations in CSF explained 26% of tau and 29% of Aβ_1–42_ variation in the AD group. The similar impact of miR-146a on these biomarkers possibly suggests a further nexus between Aβ_1–42_ and tau pathologies in AD. Another study has found elevated expression levels of miR-146a in CSF and brain regions affected by AD and also in mouse models implicating a role of miR-146a in AD pathogenesis [58, 59]. Furthermore, we did not see any significant correlations of miR-146a with tau, p-tau or Aβ_1–42_ concentrations in the control group. Hence, these findings may be specific for our AD patients.

Frigerio *et al*. reported miR-27a-3p to be significantly reduced in CSF of AD compared to controls and to correlate with tau, p-tau and Aβ_1–42_ [47]. We could not replicate the reported correlations with high tau and low Aβ_1–42_ CSF concentrations and did not observe a downregulation of miR-27a-3p in our AD samples.

Interestingly, we observed that in addition to the 6 miRNAs from set A, also potential markers such as miR-9, which did not exceed the 0.8 threshold, scored substantially higher than amyloid-beta after the reliability analysis as indicated in Fig 2A and 2B. MiR-9 is specifically enriched in the brain [60] and suggested part of a network, that indirectly regulates the APP processing, Aβ production and accumulation [61].

Moreover, we could also confirm the 9 *informative* miRNAs (miR-505-5p, miR-4467, miR-766, miR-375, miR-708, miR-3622b-3p, miR-296, miR-219 and miR-103) from set B as significant biomarkers by MANCOVA all reaching Bonferroni corrected significance. However, the low FOC in set B did not only prevent a reliability analysis but may also have reduced the property of these miRNAs to be robust biomarkers as a direct consequence. By performing discriminant analysis including candidate miRNAs of both subsets as well as in combination with CSF protein marker, we could, irrespective of FOC, demonstrate overall classification rates of 96% (miR-100, miR-375 and miR-103) and 86.4% (miR-146a and p-tau). This clearly demonstrated that already a limited number of miRNAs may be sufficient to detect AD in CSF and support our hypothesis that miRNAs could be promising and robust biomarkers for the diagnosis of neurodegenerative diseases like AD. Comparing results with those found in other body fluids, overall classification rates observed with CSF-based miRNAs are substantially higher [62].

The few biomarker screening studies [15, 47, 56, 63–65] who investigated CSF miRNA expression levels in AD did not lead to the unequivocal identification of biomarkers (S6 Dataset), in part due to problems with replicability [26, 48, 65]. Several miRNA profiling protocols for the detection of miRNAs, as reviewed in Pritchard *et al*. (2012), exist [27]. Mestdagh *et al*. (2014) suggested that differences in these protocols may explain some of the divergent results [66]. Recently, our RT-qPCR approach has been validated by Carre *et al* (2014) by using plasma samples [67]. Validation results showed that this customized method is not only sensitive and highly specific but also repeatable and accurate to detect circulating miRNAs in body fluids. According to established guidelines in the field, we favour—at least to allow a better comparability and transparency—to adhere to the MIQE guidelines or report the extent to which these guidelines were applied (S1 Dataset).

The experiments reported here demonstrate that differentially expressed miRNAs in CSF present *informative* markers that are able to detect AD compared to heterogeneous controls. However, developing microRNAs into accurate and useful tools for diagnosis of AD, will require an extensive phase of validation with multiple replication studies. This compares to the intensive work that was required to establish and approve the use of the classical protein markers tau, p-tau and beta-amyloid species in clinical routine diagnosis. These traditional CSF markers are in use in the field of dementia diagnosis for over two decades now and are far from being implemented as an easy standardized laboratory method due to pre-analytical and analytical problems that are still unsolved [68]. One can assume that this will also be an issue in miRNA based diagnostic procedures. Another negative aspect is that only a limited number of miRNAs appear to abundantly circulate in CSF. We suggest future investigations to focus on those miRNAs, like in our set A, which demonstrate high occurrence frequencies and high expression levels. A further limitation is the currently observed inter-platform variability and diversity of different experimental procedures to measure miRNA expression levels that led to inconsistencies among comparable studies (S6 Dataset) [69]. More work is required to increase data transparency (e.g. adherence to MIQE if usinq RT-qPCR) and to allow better comparisons of miRNA expression data. This is an important prerequisite on the way to establish the clinical utility of circulating miRNAs in CSF in AD diagnosis. In addition to these pre-analytical considerations, it is important that further pilot screening or candidate approach studies are based on larger patient cohorts than those reported thus far. This limitation needs to be addressed to compensate for technical and confounding variation when looking at circulating miRNAs with low expression levels [70].

However, it is advantageous that miRNAs are robust and stable in CSF and very resistant to RNAse activities that cause many problems with e.g. mRNA measurements [21]. The stability of miRNAs may greatly facilitate the standardization of sampling and detection procedures solving an issue that currently hampers the use of beta-amyloid as a biomarker for AD, which requires stringent pre-analytic sample procedures to deliver reliable results [71].

In summary, we have found putative new AD biomarkers, which display some promising attributes and face validity with view to their targets, and which, if developed into diagnostic markers, could prove to be an advantageous opportunity in clinical routines for neurodegenerative diseases such as AD. Another upcoming field is the development of miRNA treatment strategies. The identification of dysregulated miRNAs is a first step to this endeavor.

## Supporting Information

S1 Dataset MIQE checklist. E = essential, D = desired, MP = manufacturer’s protocol, N/A = not applicable.
(XLSX)

S2 Dataset MiRNAs detected in ante-mortem CSF of AD- and control group patients. N/A = not applicable, ND = not detected (Cq > 34).
(XLSX)

S3 Dataset Scatter plots of differentially regulated miRNAs in CSF of AD patients. Log2-transformed miRNA expression ratios obtained from RT-qPCR analysis are plotted for the *most reliable* (RF ≥ 0.8) miRNAs from set A: (A) miR-100, (B) miR-146a, (C) miR-1274B and the *most informative* (MoR-value d≥0.57) miRNAs from set B: (D) miR-505*, (E) miR-375, and (F) miR-103. All miRNAs were statistically confirmed by MANCOVA at Bonferroni corrected significance α = 0.05). Each data point represents one sample. For each sample, fold change in miRNA expression is calculated over its mean expression in the control group.
(TIF)

S4 Dataset In-silico predicted mRNA targets of miRNA biomarker from set A and B. MiRNA targets were predicted in silico by using the microRNA target filter tool implemented in Ingenuity Pathway Analysis (IPA) (Ingenuity Systems, www.ingenuity.com). Set = array set, target gene = predicted target, confidence = prediction confidence, pathway = related biological pathway.
(XLSX)

S5 Dataset ROC curve analysis. ROC curves for the combination of (A) miR-146a and p-tau, and (B) miR-100, miR-103 and miR-375 to separate 28 control- from 22 AD cases.
(TIF)

S6 Dataset Differentially expressed CSF miRNAs in AD. Listed are CSF miRNAs from comparable studies that were identified as significantly deregulated in AD compared to controls. MiRNAs in green indicate replicated markers and in bold novel markers that were identified in our study according to the MoR method.
(DOCX)
