# Supplementary figures and images for: MicroRNA Profiling of CSF Reveals Potential Biomarkers to Detect Alzheimer`s Disease
Source: PLoS One. 2015 May 20;10(5):e0126423. doi: 10.1371/journal.pone.0126423 (PMC4439119; doi:10.1371/journal.pone.0126423)

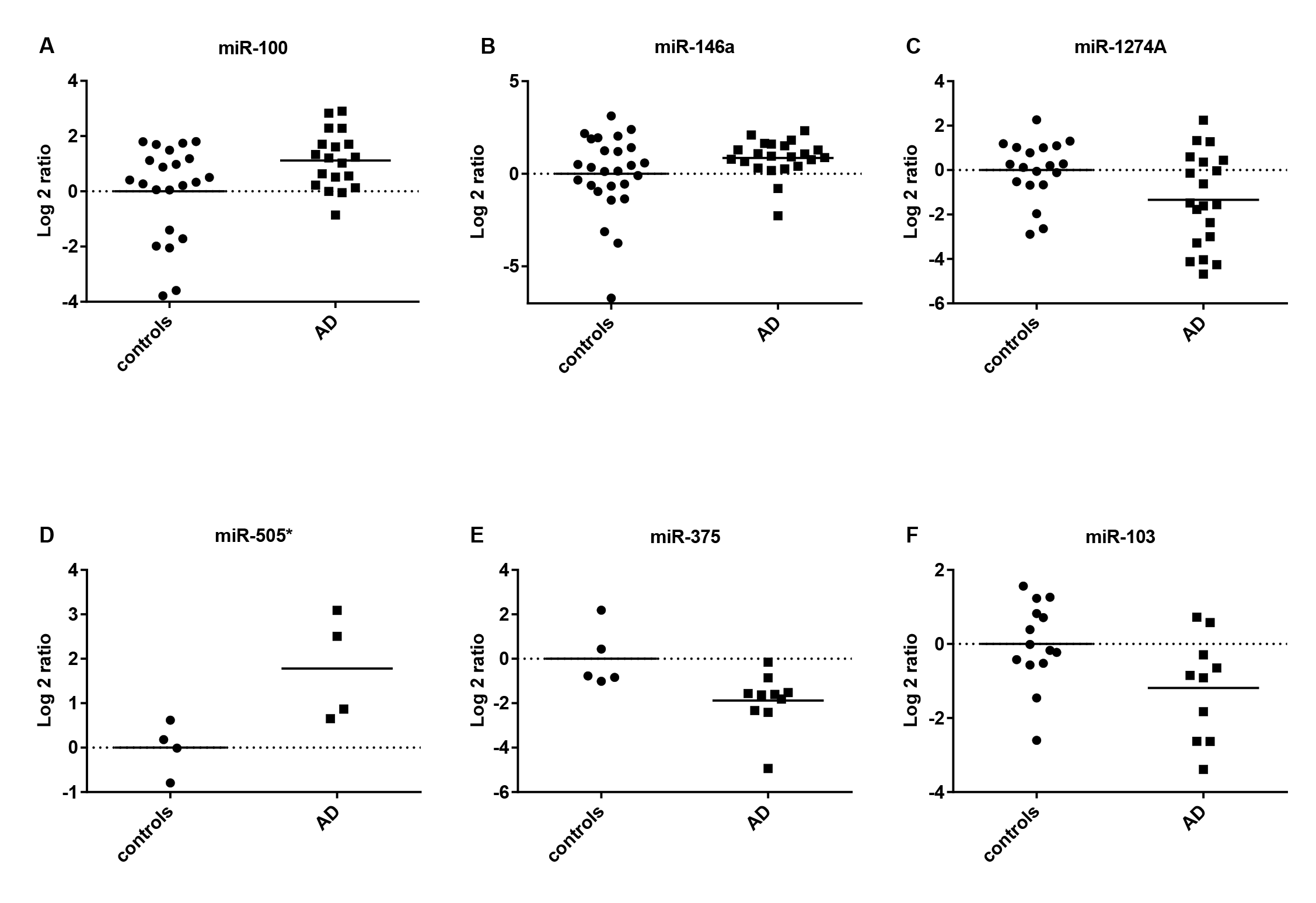

Supplement: S3 Dataset — Log2-transformed miRNA expression ratios obtained from RT-qPCR analysis are plotted for the most reliable (RF ≥ 0.8) miRNAs from set A: (A) miR-100, (B) miR-146a, (C) miR-1274B and the most informative (MoR-value d≥0.57) miRNAs from set B: (D) miR-505*, (E) miR-375, and (F) miR-103. All miRNAs were statistically confirmed by MANCOVA at Bonferroni corrected significance α = 0.05). Each data point represents one sample. For each sample, fold change in miRNA expression is calculated over its mean expression in the control group. (TIF) [file pone.0126423.s003.tif]

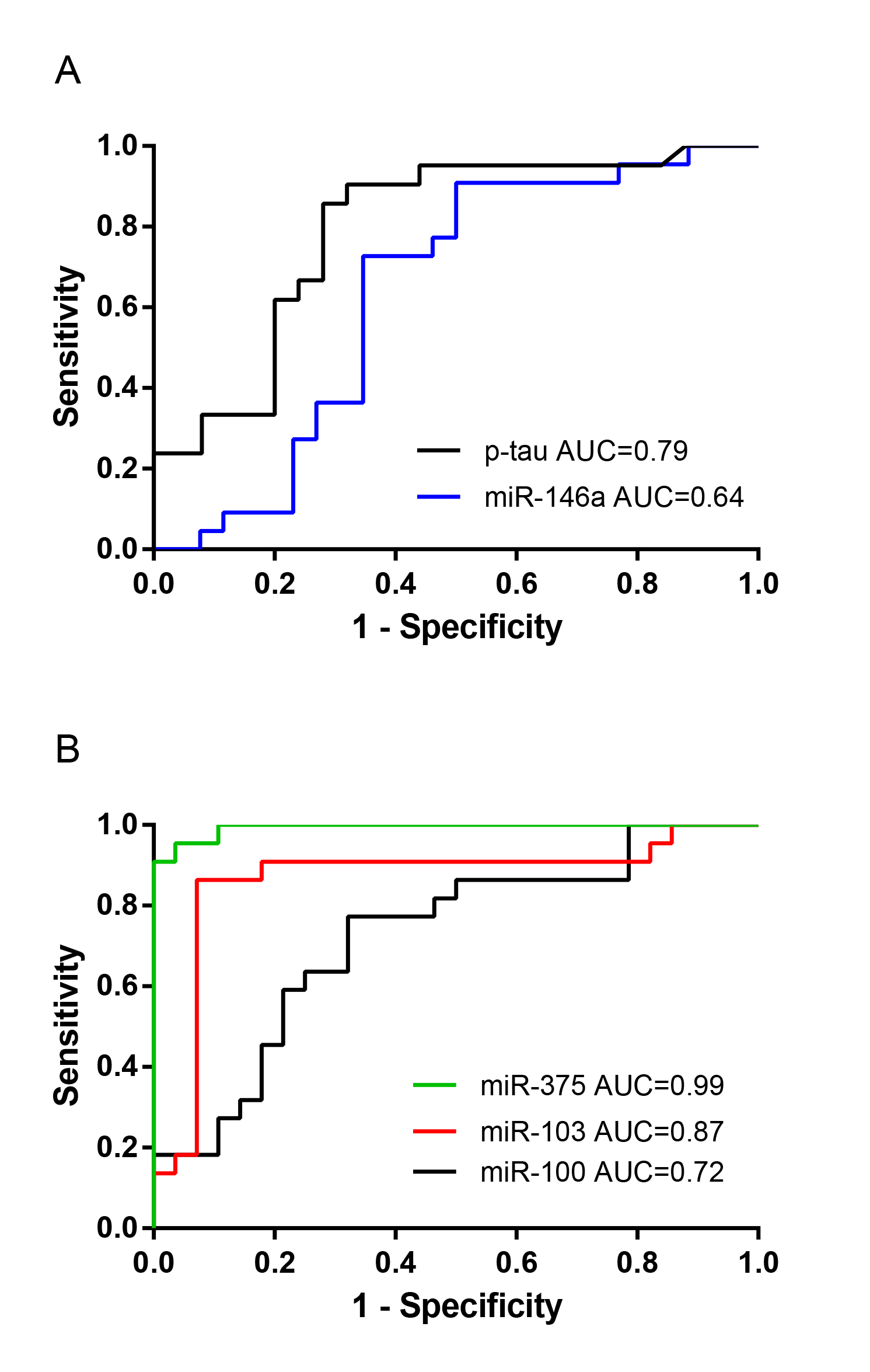

Supplement: S5 Dataset — ROC curves for the combination of (A) miR-146a and p-tau, and (B) miR-100, miR-103 and miR-375 to separate 28 control- from 22 AD cases. (TIF) [file pone.0126423.s005.tif]
