# Supplementary material for: MicroRNA Profiling of CSF Reveals Potential Biomarkers to Detect Alzheimer`s Disease
Source: PLoS One. 2015 May 20;10(5):e0126423. doi: 10.1371/journal.pone.0126423 (PMC4439119; doi:10.1371/journal.pone.0126423)
Supplement: S6 Dataset — Listed are CSF miRNAs from comparable studies that were identified as significantly deregulated in AD compared to controls. MiRNAs in green indicate replicated markers and in bold novel markers that were identified in our study according to the MoR method. (DOCX) [file pone.0126423.s006.docx]

| **miRNAs** | **Changes** | **Cohort** | **Method** | **References** |
| --- | --- | --- | --- | --- |
| let-7f, miR-105, -125a, -135a, -138, -141, -151, -186, -191, -197, -204, -205,- 216, -302b, -30a-5p, -30a-3p, -30b, -30c, -30d, -32, -345, -362, -371, -374, -375, -380-3p, -429, -448, -449, -494, -501, -517, -517b, -518b, 518f, 520a*, 526a | up-regulated | 10 patients  10 controls | RT-qPCR  (TaqMan) | Cogswell 2008 |
| miR-10a, -10b, -125, -126*, -127, 142-5p, -143, -146b, -154, -15b, -181a, -181c, -194, -195, -199a*, -214, -221, -328, -422b, -451, -455, -497, -99a | down-regulated |  |  |  |
| miR-27a-3p | down-regulated | 8 patients  8 controls | RT-qPCR  (SYBR green) | Frigerio 2013 |
| miR-9, -125b, -146a, -155 | up-regulated | 6 patients  6 controls | microarray/  northern blot | Alexandrov 2012 |
| miR-34a, -125b, -146a, -9 | down-regulated | 10 patients  10 controls | RT-qPCR  (TaqMan) | Kiko 2014 |
| miR-29a, -29b | up-regulated |  |  |  |
| miR-146a | down-regulated | 20 patients  20 controls | RT-qPCR  (TaqMan) | Müller 2014 |
| **miR-100**, -146a, **-296**, **-3622b-3p**, **-4467**, **-505***, **-766** | up-regulated | 22 patients  28 controls | RT-qPCR  (TaqMan) | Reported in our study |
| **miR-103**, **-219**, **-335**, -375, **-708**, **-1274A**, **-4449**, -**4674** | down-regulated |  |  |  |
